# Migration and "Low-Skilled" Workers in Destination Countries

**DOI:** 10.1371/journal.pmed.1001043

**Published:** 2011-06-07

**Authors:** Joan Benach, Carles Muntaner, Carlos Delclos, María Menéndez, Charlene Ronquillo

**Affiliations:** 1 Health Inequalities Research Group, Employment Conditions Knowledge Network (GREDS-EMCONET), Universitat Pompeu Fabra, Barcelona, Spain; 2 CIBER Epidemiología y Salud Pública (CIBERESP), Barcelona, Spain; 3 Bloomberg Faculty of Nursing and Dalla Lana School of Public Health, Division of Social and Behavioural Health Sciences, University of Toronto, Toronto, Ontario, Canada; 4 Department of Political and Social Sciences, Universitat Pompeu Fabra, Barcelona, Spain; 5 Department of Occupational Health, Comissions Obreres (CC.OO.), Barcelona, Spain; 6 Bloomberg Faculty of Nursing, University of Toronto, Toronto, Ontario, Canada

## Abstract

In the fourth article in a six-part *PLoS Medicine* series on Migration & Health, Joan Benach and colleagues discuss the specific health risks and policy needs associated with migration in destination countries, especially for low-skilled and illegal migrant workers.

**Summary Points**

- The last few decades have seen substantial increases in the migration of migrant workers, raising concerns about the health implications of these movements.
- In destination countries, migrant workers often fill undesirable, low-skill positions characterized by flexibility, insecurity, precarious employment, and long working hours with low pay.
- Undocumented or “illegal” migrants are especially vulnerable to exploitation since they fear job loss, incarceration, and deportation.
- Urgent health issues to be addressed among migrant workers include occupational safety, injury prevention, work-related diseases, barriers to accessing health services, and the associated health risks for their families and communities, in addition to discrimination and exploitation.
- Governments, unions, and international organizations should collaborate to implement fair labour standards for both legal and illegal labourers that are on par with citizen workers, standardise labour migration policies, and provide legal support for undocumented labourers to help eradicate human trafficking and other forms of extreme labour exploitation.

**This is one article in a six-part** ***PLoS Medicine*** **series on Migration & Health**.

## Introduction

Over the last few decades, increases in international migration have transformed the lives of hundreds of millions of people around the globe. Since 1975, the number of international migrants has more than doubled, with most living in Europe (56 million), Asia (50 million), and North America (41 million). Nowadays, 3.1% of the world population resides in a country other than where they were born [1], and when one includes visitors on business or personal trips, roughly one million people move between the high-income and mid- and low-income countries each week [1].

Although people cite many reasons for why they move from their home country to another, there is little doubt that a large increase in international migration has been driven by economic globalisation. Roughly half of all international migrants are economically active migrant workers [2]. Generally, these workers who move from low-income to middle- and high-income countries are searching for ways to provide for their families and to escape unemployment, war, or poverty in their countries of origin [2]. Through their high-skill and low-skill labour, migrant workers contribute to growth and development in destination countries by creating new demands for housing and other products and services. Changes in global production systems, along with demographic factors and labour market dynamics in countries of origin, have relegated millions of workers from poor countries to serving as a source of cheap, flexible, low-skill labour and direct and as indirect taxpayers in wealthier countries. Meanwhile, labour markets in destination countries do not often provide workers who are willing to accept precarious employment with long working hours for low pay [3].

In destination countries, migration has important implications for public health and health care. While high-skilled migrant workers may suffer from some potential risks, they also receive various benefits. For instance, the negative effects of migration on health tend to spare migrants of high socioeconomic position [4], whereas studies have shown that low-skilled migrant workers, for example, are at risk of contracting diseases unknown to their region of origin [5]. The health of migrant workers is also affected by their exploitation. That is, migrant workers often serve as the low-skill labour force that fills the “3-D” jobs (“dangerous, dirty and degrading”) that national workers are reluctant to perform, despite often being over-qualified for these positions [6]. Thus, migrants are more often exposed to potentially health-damaging work environments than native workers.

The aim of this article is to examine the relationship between migration, employment, and health in destination countries by focussing on the employment and working conditions experienced by low-skilled migrant workers. We discuss the policy implications stemming from these complex relationships.

## Work and Health of Migrant Workers in Destination Countries

In most destination countries, migrant workers are found in the agricultural, food processing, and construction sectors of the economy, in semi-skilled or low-skill manufacturing jobs, and in low-wage service jobs. Foreign-born workers are vulnerable to coercion into precarious employment conditions within those sectors as a result of their irregular and undocumented legal status [7]. A large number of migrant workers work on the lowest rung of the destination country's employment ladder in low-skill day labour, which holds no guarantee of future work and mainly employs recently arrived immigrants [7]. While the characteristics and size of populations of migrant day labourers have been difficult to establish, the number of migrants in an irregular situation was estimated by the International Labour Organization (ILO) in 2006 to be roughly 20–30 million globally [8]. Migrant status can be an important source of global occupational health inequalities, regardless of whether the individual is considered low-skilled or highly skilled. Recent reviews on employment and health inequalities suggest that migrant status is a key cross-cutting mechanism linking employment and working conditions to health inequalities through diverse exposures and mechanisms [3],[9].

Migrant day labourers who cannot obtain work permits are especially vulnerable to exploitation, since they fear job loss, incarceration, and deportation; they can be hired at extremely low wages and are often underpaid or not paid at all [10]. Furthermore, migrant day labourers are often exposed to a variety of work-related hazards (such as chemicals, pesticides, dust, and other toxic substances) without proper protective equipment, compensation insurance, or on-the-job training [7],[11]. The health issues to be considered for these workers are many including, among others, occupational safety and injury prevention, work-related diseases, and barriers to accessing mental health services. For instance, because of the high-risk conditions in which many migrant day labourers work, injury is an ever-present threat. Worldwide, ILO has estimated that migrant day labourers experience 335,000 accidents per year in the most dangerous of industries—agriculture, mining, and construction [12]. In the US, Valenzuela et al. reported that one in five migrant day labourers has suffered an injury, and agricultural hazards accounted for 7.4% of these work-related deaths [13].

Low-skill migrant workers tend to experience more serious situations of discrimination and exploitation in destination countries than native workers. In particular, bonded labour most directly affects migrant women working in insecure informal economy jobs [14],[15]. As the most vulnerable workers in destination countries, female migrant labourers work primarily in retail, domestic work, or consumer services [16]. Foreign-born women in irregular jobs can become trapped into smuggling or servitude by migration agents, criminal organisations, or sex traffickers. It has also been shown that women do not resort to smugglers as often as men, which is in fact a result of their knowledge of how much more hazardous smuggling is for them than for men [17]. Another important issue is the enormous health risks to migrant women and children. A systematic review found that pregnant migrant women are at risk for worse pregnancy outcomes, particularly in destination countries that do not have strong policies for the integration of migrant communities [18]. For children whose parents are migrant workers, being excluded from medical services and the educational system in the destination country can lead to serious mental and physical problems [19],[20]. The effects of job insecurity on psychological distress and overall health constitute yet another burden for migrant workers and their families [21],[22].

While most studies of migrant workers in destination countries refer to occupational injuries, many have also analysed and found evidence of a large number of work-related social problems, including social exclusion, lack of health and safety training, fear of reprisals for demanding better working conditions, concealing their need for medical care from employers, lack of knowledge regarding their rights as workers, linguistic barriers that minimize the effectiveness of training, and difficulty accessing care and compensation when injured [7]. Their unstable working conditions and associated social isolation also induce a risk of poor mental health such as chronic stress, anxiety, and depression [23]. Although a few of these labourers, when injured, receive medical treatments covered by employer-sponsored insurance, the vast majority obtain no treatment at all [24].

The immediate future does not bode well for the health of migrant workers, as the global economic recession is likely to heavily impact migrants, who constitute the most vulnerable and deprived segment of the workforce [17],[25]. Previous economic downturns, such as the East Asian economic crisis in the 1990s, were found to aggravate already negative conditions for these deprived and vulnerable workers [26], and it is reasonable to suggest that the current global economic crisis has similarly aggravated their harsh employment conditions and their health [27].

## Policy Considerations

Policies to achieve better employment and working conditions among immigrants in destination countries require the implementation and evaluation of programs outside the health care sector. To identify what works across different historical and political contexts is an urgent and essential task in addition to a critical examination of existing policies as determinants of health for immigrants.

Arguably, institutional policies in destination countries also contribute to imposing further restrictions on migrant workers that can directly or indirectly affect their health. One example is Canada's Live-In Caregiver Program, often criticised for facilitating exploitation of migrant women. The live-in requirement and compulsory completion of a minimum number of hours to become eligible for permanent residency, all the while providing no monitoring or regulation of work conditions or payment, are features of this program that leave participating migrants particularly vulnerable [28],[29]. Often, these features result in newly arrived immigrants without supportive networks or resources being overworked, completing domestic duties that are not within the scope of caregiving, being underpaid, and facing a substantial imbalance of power that leave them at the hands of their employers for fear of not completing the program requirements and having to face deportation [29],[30]—all factors that impact both somatic and mental health. Such policies by host countries need to be critically evaluated in terms of their consequences, whether these are intended or not.

For migrant workers, the excessive risk of injury and disease linked to dangerous employment and working conditions in their destination countries is a global phenomenon. To reduce employment-related health inequality for these labourers in their countries of destination, future legislation backed by research, evaluation, and monitoring, on a country-by-country basis, is urgently needed so that better understanding is gained of (1) what true magnitude, mechanism, and pathways underlie the relation between migrant health inequality and employment conditions, (2) how health effects vary according to the hosting county's labour regulations and policy, and (3) how a global economic recession amplifies health inequality for legal and illegal migrant day labourers [31]. To ensure their health and safety, governments, unions, and international organizations should collaborate to implement fair labour standards by (1) administering adequate supervision, safety training, health surveillance, and work-related insurance for legal and illegal labourers that are on par with citizen workers, and (2) standardising labour migration policies while instituting legal support for these undocumented labourers to help eradicate human trafficking and other forms of extreme labour exploitation [3],[32].

Immigration policies of sending countries similarly require closer scrutiny, particularly regarding weighing the balance of positive and negative consequences of migration for the individual versus the country. Although governments may present the premise of migration as positive to the public, it is important to scrutinize the motives behind pro-migration policies in addition to other policies in place, if any, that provide support and protection for migrant workers. For example, return migration (i.e., return of migrants to the home country) is often argued to be a positive consequence of migration. Yet, it is important to consider that reintegration of migrant workers into their country of origin with skills acquired through their migration experiences are often complicated by hindrances such as high unemployment [3]. The need to address such issues by the governments of sending nations become especially poignant during periods of economic downturn in host countries, when migrants may have to return “home.” The loss of economic and other investments in migration, coupled with unemployment and the inability to re-enter the home country's labour market, place returning workers and their families at risk of poverty and ill health [3].

Return migration is less likely to occur in countries where migrants enjoy secure and stable residency [17]. Examples of the return migration “failure” have been observed in the Philippines' outflow of nurses, the majority of whom do not return to their home country [33],[34]. So far, strategies such as providing financial incentives for return migration, which have been introduced in some countries (e.g., in the Czech Republic and Spain), have had limited success [17]. As programs like the Migration for Development in Africa (MIDA) are implemented to encourage and facilitate return migration, a consideration of non-economic factors influencing return (e.g., political instability in the home country) is crucial for understanding the potential for success of such programs.

Although specific mechanisms are poorly understood, current evidence shows that the employment and working conditions faced by most migrant workers are dangerous to their health. Ultimately, more global health surveillance and socio-epidemiologic analyses of migration will be needed to render employment conditions prominent in migration policy.

## Abbreviations

ILO: International Labour Organization
